# Screening for proteinuria in ‘at-risk’ patients with spinal cord injuries: lessons learnt from failure

**DOI:** 10.1186/1754-9493-8-25

**Published:** 2014-06-09

**Authors:** Subramanian Vaidyanathan, Kottarathil Abraham Abraham, Gurpreet Singh, Bakul Soni, Peter Hughes

**Affiliations:** 1 Regional Spinal Injuries Centre, Southport and Formby District General Hospital, Town Lane, Southport PR8 6PN, UK; 2 Department of Renal Medicine, Southport and Formby District General Hospital, Town Lane, Southport PR8 6PN, UK; 3 Department of Urology, Southport and Formby District General Hospital, Town Lane, Southport PR8 6PN, UK; 4 Department of Radiology, Southport and Formby District General Hospital, Town Lane, Southport PR8 6PN, UK

**Keywords:** Spinal cord injury, Proteinuria, Kidney, Renal calculi

## Abstract

Spinal cord injury patients may develop proteinuria as a result of glomerulosclerosis due to urosepsis, hydronephrosis, vesicoureteric reflux, and renal calculi. Proteinuria in turn contributes to progression of kidney disease. We report one paraplegic and two tetraplegic patients, who developed recurrent urine infections, urinary calculi, and hydronephrosis. These patients required several urological procedures (nephrostomy, cystoscopy and ureteric stenting, ureteroscopy and lithotripsy, extracorporeal shock wave lithotripsy). These patients had not received antimuscarinic drugs nor had they undergone video-urodynamics. Proteinuria was detected only at a late stage, as testing for proteinuria was not performed during follow-up visits. Urine electrophoresis showed no monoclonal bands in any; Serum glomerular basement membrane antibody screen was negative. Serum neutrophil cytoplasmic antibodies screen by fluorescence was negative. All patients were prescribed Ramipril 2.5 mg daily and there was no further deterioration of renal function.

Spinal cord injury patients, who did not receive antimuscarinic drugs to reduce intravesical pressure, are at high risk for developing reflux nephropathy. When such patients develop glomerulosclerosis due to recurrent urosepsis, renal calculi, or hydronephrosis, risk of proteinuria is increased further. Take home message: (1) Screening for proteinuria should be performed regularly in the ‘at-risk’ patients. (2) In the absence of other renal diseases causing proteinuria, spinal cord injury patients with significant proteinuria may be prescribed angiotensin-converting enzyme inhibitor or angiotensin-II receptor antagonist to slow progression of chronic renal disease and reduce the risk of cardiovascular mortality.

## Background

A cross-sectional analyses of Veterans with spinal cord injury and disorders across all VA facilities of USA in 2006.revealed that 1 in 3 Veterans with had chronic kidney disease [1]. The United Kingdom national guidelines on ‘chronic spinal cord injury: management of patients in acute hospital settings’, state that urinary assessment should include review of voiding method and pattern; 24-hour voided volume chart; post-void residual volume (by catheter or bladder scan), if voiding on urge or by reflex; urinary microscopy and culture, if symptoms or signs of local or systemic infection [2]. There is no mention of testing for proteinuria. The Consortium for Spinal Cord Medicine consisting of seventeen organizations, including the Paralyzed Veterans of America developed clinical practice guidelines in spinal cord medicine [3]. Bladder management for adults with spinal cord injury recommends a urologic evaluation every year, although there is no consensus among doctors on the frequency this type of exam should be performed or the range of tests that should be included. The important components of the urologic evaluation are an assessment of both the upper and lower tracts. Upper tract evaluations include tests that evaluate function, such as renal scans and tests that evaluate anatomy, such as ultrasound, CT scans, and intravenous pyelograms (IVP). Renal scans are frequently used to screen the upper tract because they are not user dependent, do not have a risk of allergic reactions, do not require a bowel preparation, and cause much less radiation exposure than a CT scan or IVP. Lower tract evaluations include urodynamics to determine bladder function, cystograms to evaluate for vesicoureteral reflux, and cystoscopy to evaluate bladder anatomy. Thus testing for proteinuria does not appear to be a mandatory investigation. In North West Regional Spinal Injuries Centre in Southport, UK, blood tests (urea, creatinine, and eGFR), and ultrasound examination of urinary tract are carried out during follow-up of spinal cord injury patients. No test is done to detect proteinuria during follow-up.

Wall and associates [4] found following independent predictors for the development of proteinuria in spinal cord injury patients by using logistic stepwise multiple linear regression analysis: (1) Use of chronic indwelling bladder catheters. (2) Number of decubitus ulcer procedures. (3) Older age. (4) Patients with co-morbidities such as diabetes mellitus, hypertension.

Proteinuria is likely in spinal cord injury patients, who did not receive antimuscarinic drugs to reduce intravesical pressures and therefore, are at high risk for developing reflux nephropathy and consequently proteinuria [5]. When spinal cord injury patients develop glomerulosclerosis as a result of recurrent urosepsis, hydronephrosis, and renal calculi, they are likely to manifest proteinuria. We report these patients in whom, we failed to detect proteinuria in early stage. We wish to share our experience so that spinal cord physicians are made aware of the need to look for proteinuria in ‘at- risk’ patients and similar medical errors do not happen again.

## Case scenarios

### Case 1

**Figure 1:**
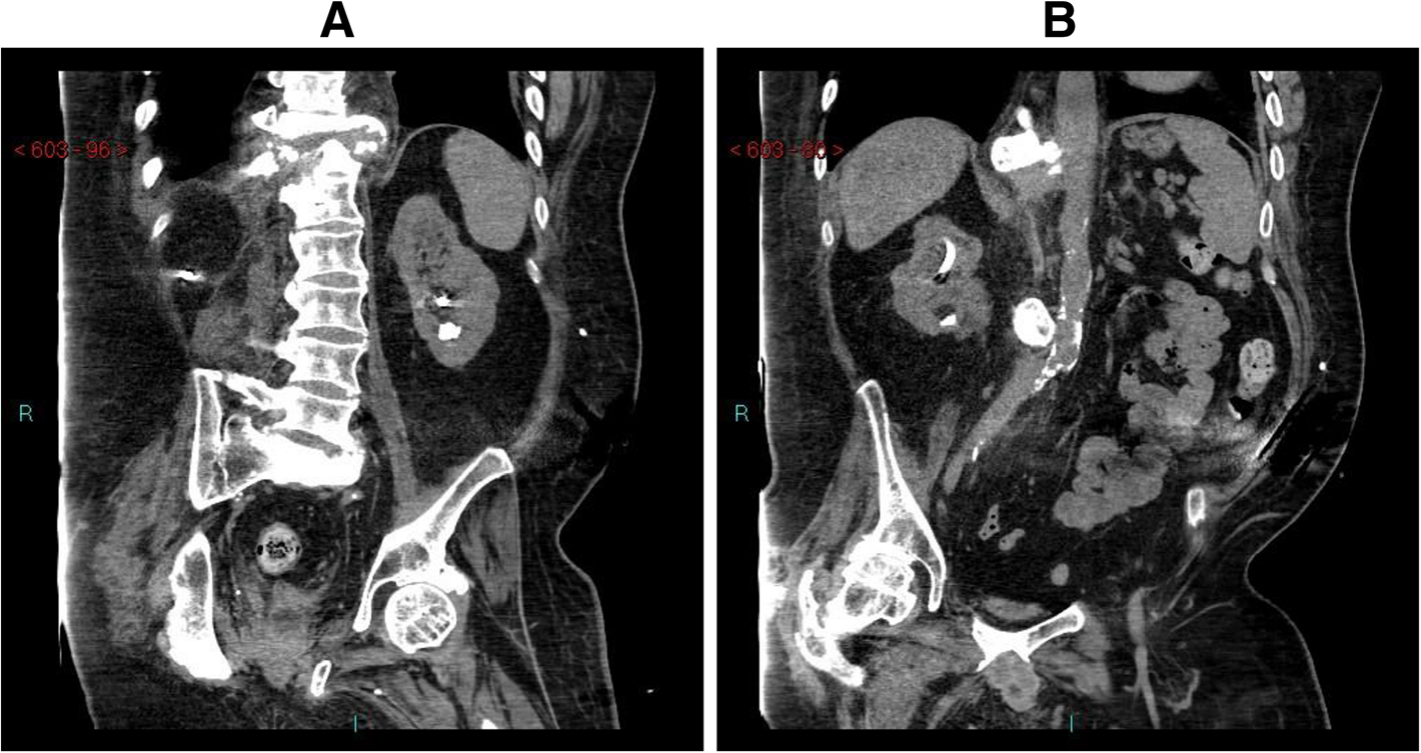
**Case 1: Computed Tomography of kidneys, coronal section. (A)** Left kidney. Calculus in lower calyx; left nephrostomy in place; renal outline is irregular due to cortical scarring. **(B)** Right kidney: stone in lower pole; right nephrostomy in place; cortical margin is irregular. Calcification of aorta is seen.

An 18-year-old, British male, while attending a party, was held by the neck and thrown out in 1981. He fell on his face and found that he could not move his limbs. This patient had sustained C-5 tetraplegia. X-ray of cervical spine revealed C-6/C-7 dislocation. During rehabilitation, this patient had indwelling urethral catheter drainage. He developed several episodes of urine infection and received multiple courses of Ampicillin, Gentamicin, and Amikacin. Cystogram revealed right vesicoureteric reflux. In 1982, division of external urethral sphincter was performed. He was prescribed Phenoxybenzamine 10 mg three times a day. Despite this, he had high residual urine volume and he developed urine infections. Cystogram revealed persistence of right vesicoureteric reflux. In 1983, bladder neck resection was performed. He was prescribed Distigmine and penile sheath drainage was tried. In hindsight, Distigmine could have predisposed high pressure voiding and reflux nephropathy. This patient developed recurrent urine infections and repeated attacks of orchitis. In 2001, stones in right renal pelvis were treated by extracorporeal shock wave lithotripsy. Subsequently, this patient felt unwell and had rigors. Ultrasound examination revealed left hydronephrosis and a calculus at the ureteropelvic junction. Left ureteric stenting was done followed by extracorporeal shock wave lithotripsy of left renal calculus. Later, this patient developed stone in left ureter; ureteric stenting was done in another hospital. Computed tomography revealed that the stone was lying outside left ureter, anterior to left psoas muscle. The ureteric wall was thickened and ureteric lumen was narrow. Extracorporeal shockwave lithotripsy of left renal calculus was performed. Subsequently, the ureteric stent could not be removed because of encrustations over the proximal coil. Extracorporeal shock wave lithotripsy of encrustations over proximal coil of ureteric stent was performed. The ureteric stent was then removed. A month later, this patient developed temperature and shivering. Ultrasound revealed moderate left sided hydronephrosis with mildly reflective urine in the collecting system, suspicious of infection. Left ureteric stenting was done and the patient received Gentamicin. During follow-up, the stent was removed, but a new stent could not be inserted. He developed temperature and rigors. Left nephrostomy was performed in 2009. Nephrostomy tube was being changed every 6–8 weeks. In 2012 this patient became unwell. Ultrasound revealed hydronephrotic right kidney with stone in pelviureteric junction. Right nephrostomy was performed. Extracorporeal shock wave lithotripsy of right renal calculus was carried out. Computed tomography revealed residual stone fragments and cortical scarring (Figure 1) Results of laboratory investigations are given in Table 1.

**Table 1:** Results of laboratory investigations of Case 1

▪	Urea: 4.9 mmol/L
▪	Creatinine: 52 umol/L
▪	Haemoglobin: 152 g/L
▪	HbA1c: 35 mmol/mol
▪	Serum total protein: 69 g/L
▪	Albumin: 41 g/L
▪	Urine microalbumin: 239.1 mg/L
▪	24-hours urine protein: 0.32 g/24 hours (0.10 g/L) from left kidney and 0.46 g/24 hours (0.19 g/L) from right kidney.
▪	Urine electrophoresis: No monoclonal bands were detected.
▪	Serum Glomerular Basement Membrane antibody screen: Negative
▪	Serum Neutrophil Cytoplasmic Antibody screen by florescence: Positive p-ANCA not MPO. The significance of this antibody is unknown.
▪	Serum Anti-Proteinase (c-ANCA): Negative
▪	Serum Anti-Myeloperoxidase (p-ANCA): Negative

He was prescribed Ramipril 2.5 mg daily. There was no side-effect to Ramipril. This patient continues to have bilateral nephrostomy drainage. Blood pressure: 144/62 mm Hg. He comes to spinal unit twice a week for change of nephrostomy dressings.

### Case 2

**Figure 2:**
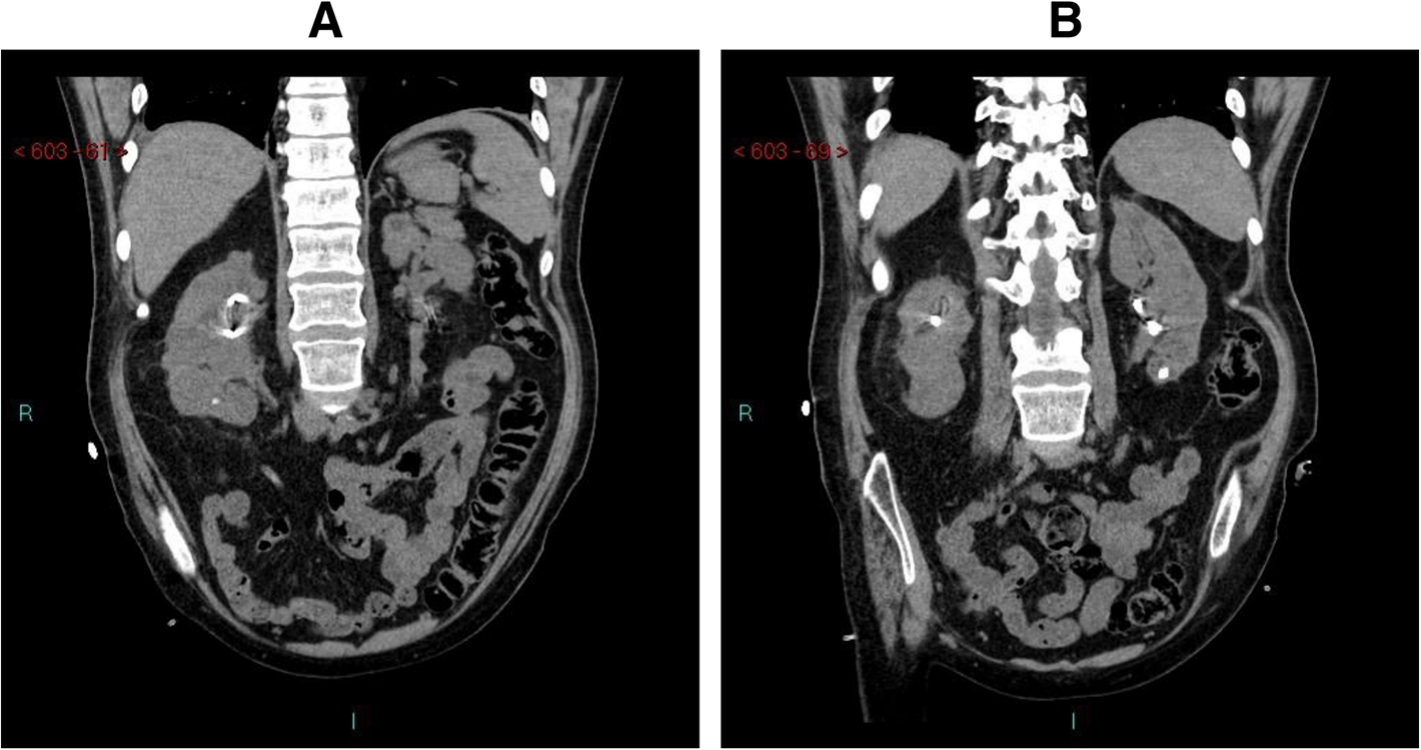
**Case 2: Computed Tomography of kidneys, coronal view. (A)** Right kidney: tiny calculus in lower pole; nephrostomy is in place. Outline of kidney is irregular due to cortical scarring. **(B)** Left kidney calculus in lower pole; nephrostomy is seen in place. Renal outline is irregular.

A British white male sustained T-4 complete paraplegia in a motor bike accident in 1988. The lower urinary tract had been managed by penile sheath drainage, He was advised intermittent catheterisations and use oxybutynin bladder instillations; but this was not continued. Video-urodynamics was not performed Blood urea: 3.0 mmol/L. Creatinine: 47 umol/L. In 1995, this patient developed vomiting and temperature. Blood urea: 22.3 mmol/L; creatinine: 299 umol/L. Ultrasound of kidneys revealed marked hydronephrosis of right kidney and minor hydronephrosis of left kidney. nephrostomy drainage of both kidneys was performed in March 1995. In April 1995, cystoscopy and bilateral ascending ureterogram were performed. Right ascending ureterogram revealed partial pelviureteric junction obstruction. A JJ stent was inserted. Left ascending ureterogram revealed a stone just below pelviureteric junction obstruction. Electrohydraulic lithotripsy was carried out; stone was partially fragmented; a 7 French JJ stent was inserted. In June 1995, blood urea was 4.2 mmol/L; creatinine: 70 umol/L. Right ureteric stent was removed. Extracorporeal shock wave lithotripsy of left ureteric stone was performed. In September 1995, left ureteric stent was removed. In November 1995, both nephrostomy tubes were removed. In 1998, intravenous urography revealed right hydronephrosis. Anderson Hine’s pyeloplasty was performed for right pelviureteric junction obstruction. In 2000, pus was pouring out of left nephrostomy scar. Computed tomography revealed left mid-pole renal abscess with extension to perinephric abscess. This patient improved with antibiotic therapy (gentamicin and metronidazole). In 2005, this patient developed fever with rigors he developed multi-organ failure requiring ventilation and noradrenaline infusion. Ultrasound scan revealed bilateral hydronephrosis with multiple stones in left kidney. Bilateral percutaneous nephrostomy were carried out. X-ray of chest revealed generalized infiltration of both lung fields. Tracheostomy was performed. With Tazocin therapy, he improved. Extracorporeal shock wave lithotripsy of left renal calculi was carried out. In 2006, balloon dilatation of right pelviureteric junction was performed. Left ureteric catheterization was not possible; Terumo guide wire could be passed through left ureteric orifice for one cm only. A stent was introduced antegrade in left ureter and nephrostomy was removed. In 2007, Right nephrostomy and left ureteric stent were replaced at regular intervals. In 2011, ultrasound examination revealed the right kidney measuring 9.4 cm with diffuse cortical scarring; cortical thinning was noted. Left kidney was grossly hydronephrotic with diffuse cortical thinning. MAG-3 renogram, performed in 2011, revealed relative function of 24% by left kidney compared with 76% for the right kidney. Uptake and drainage of left kidney had deteriorated from the earlier study with a persistent obstructive pattern. Drainage of right kidney was impaired. The findings were consistent with deterioration in cortical function of the left kidney. In 2012, this patient developed sepsis following blockage of right nephrostomy. Right nephrostomy was changed; about 200 ml of thick purulent fluid was drained. Left nephrostomy was performed two weeks later. Subsequently, left ureteric stent was removed. In 2013, computed tomography revealed tiny calculi in mid and lower pole of right kidney; 10 mm calculus at the lower pole of left kidney; cysts in left kidney; cortical scarring in both kidneys (Figure 2).

Urea: 6.9 mmol/L. Creatinine: 99 umol/L. Haemoglobin: 144 g/L. eGFR: 67 ml/min/1.73 m. Cholesterol: 7.8 mmol/L; Triglycerides: 2.4 mmol/L; LDL Cholesterol: 5.5 mmol/L; Cholesterol/HDL Ratio: 7. In February 2013, Right nephrostomy: Urine protein: 2.31 g/L; 24 hours urine protein: 1.59 g/24 hours. Left nephrostomy urine protein: 0.73 g/L; 24 hours urine protein: 0.42 g/24 hours. In October 2013, urine from left nephrostomy: Urine protein: 2.10 g/L. Protein:creatinine ratio: 488.4 mg/mmol. Right nephrostomy: 0.61 g/L Urine Protein: creatinine ratio: 112.96 mg/mmol. Results of additional tests are given in Table 2.

**Table 2:** Results of blood tests of Case 2

▪	Serum Total Protein: 77 g/L; Albumin: 41 g/L.
▪	Serum protein electrophoresis: No abnormal bands detected.
▪	Serum immunoglobulins:
▪	Serum Immunoglobulin G: 18.09 g/L (reference range: 6.0-16.0)
▪	Serum Immunoglobulin A: 4.14 g/L (reference range: 0.8 – 2.8)
▪	Serum Immunoglobulin M: 2.08 g/L (reference range: 0.5 – 1.9). Polyclonal increase of immunoglobulins was associated with infection, liver disease, or various connective tissue diseases.
▪	Serum Connective Tissue Diseases screen: Negative (tested for U1RNP, SS-ARo (60 kDa, 52 kDa), SS-B/La, Centromere B, Scl-70, Jo-1, Fibrillarin, RNA Pol III, Rib-P, PM-Scl, and PCNA, Mi-2 proteins and Sm proteins and dsDNA).
▪	Serum Glomerular Basement Membrane Screen: Negative. GBM Quantification: less than 0.8 Elisa units (0–6.9).
▪	Serum Neutrophil Cytoplasmic Antibody Screen by fluorescence: Negative.
▪	Serum Anti-Proteinase 3 (c-ANCA): Negative 0.2 IU/ml (0–1.9).
▪	Serum Anti-Myeloperoxidase (p-ANCA): Negative (less than 0.2 (0–3.4).

In view of the risk of progression of chronic kidney disease and the need to lower the proteinuria to protect his cardiovascular risk, this patient was prescribed Ramipril 1.25 mg a day and kidney function was checked a week later. Plan was to monitor the patient and if he developed any symptom due to low blood pressure, then the medication would have to be stopped. He was also prescribed Atorvastatin 10 mg daily. Three weeks later, cholesterol level decreased to 4.5 mmol/L; LDL cholesterol level decreased to 3.0 mmol/l. This patient did not develop side-effects to Ramipril; the dose of Ramipril was increased to 2.5 mg daily. Blood pressure was 119/70 mm Hg. This patient continues to have bilateral nephrostomy drainage. He is currently employed as Information Technologist in the local government council. He visits spinal unit once a week for change of nephrostomy dressings.

### Case 3

**Figure 3:**
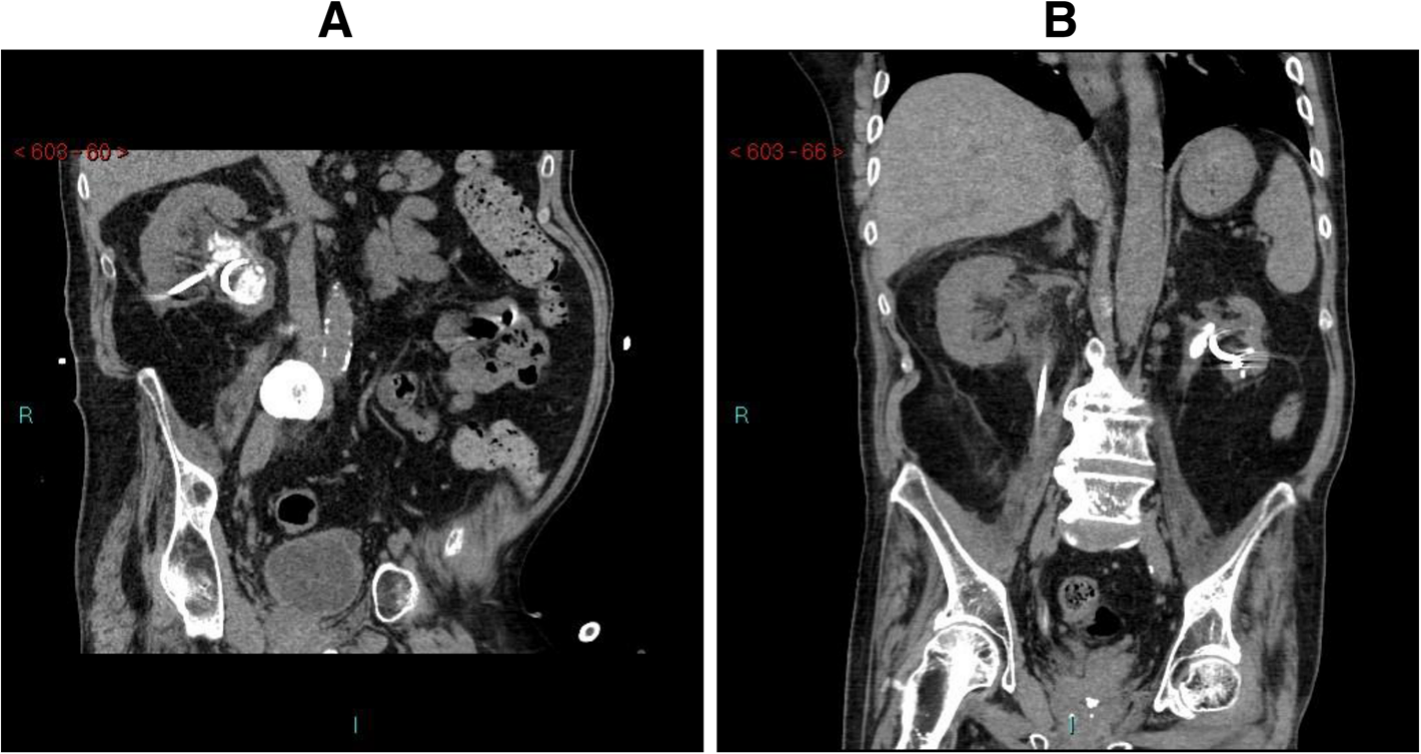
**Case 3: Computed Tomography of kidneys, coronal view. (A)** right kidney: nephrostomy in place; several calculi in renal pelvis, and calcification in aorta. **(B)** stent in right ureter; nephrostomy in left kidney; stone in left renal pelvis; and left kidney is atrophic.

A 37-year-old British male, in the year 1987, dived into the sea, and was found floating on the water face down. He was brought out of the water and taken to Emergency. Clinical examination revealed complete tetraplegia at C-6 level. Division of external urethral sphincter at 12 o’ clock position from just below bladder neck to bulb, dividing the sphincter completely, was performed in 1991. This patient managed his bladder by penile sheath along with oral Prazosin 500 micrograms twice a day. In 1996, this patient started having intermittent catheterisations twice a day. This patient was not prescribed antimuscarinic drugs and status of neuropathic bladder was not assessed by video- urodynamics. In 2000, during routine follow-up, calculus in upper pole of left kidney was detected. In 2001, ultrasound revealed calculi in mid- and lower poles of left kidney. In 2003, MAG-3 renogram showed relative function of left kidney to be 16% and 84% in right kidney. In 2004, left ureteric stenting was done and stone in urinary bladder was treated by electrohydraulic lithotripsy. Extracorporeal shock wave lithotripsy of left renal calculus was carried out and follow-up X-ray of kidney revealed complete clearance. In 2008, this patient developed severe urine infection. Computed tomography of kidneys revealed a 7.6 cm × 7 cm perinephric collection, postero-inferior to the mid and lower pole of right kidney. There was a 2 cm staghorn calculus in the renal pelvis. Left kidney appeared atrophic with multiple cortical scarring and at least two calculi in renal pelvis. Under CT guidance, abscess located in right perinephric region was drained. An 8 French pigtail catheter was inserted to drain the pus. Microbiology of pus revealed growth of *Enterococcus faecalis, Streptococcus milleri* and mixed anaerobes. After antibiotic therapy, a stent was inserted in right ureter. Extracorporeal shockwave lithotripsy was performed, which resulted in complete fragmentation of stones in right kidney. Then right ureteric stent was removed. In 2009, this patient developed stones in left kidney, and renal calculi were treated by extracorporeal shock wave lithotripsy. In 2011, this patient developed bilateral renal calculi. Extracorporeal shock wave lithotripsy of right renal calculi was carried out. In 2012, this patient became unwell. Ultrasound revealed marked hydronephrosis of left kidney. Left nephrostomy was performed. Extracorporeal shock wave lithotripsy of left renal calculi was carried out. He developed left ischial pressure sore and the sore was repaired under general anaesthesia in 2012. In 2013, multiple calculi were detected in right kidney. Subsequently, this patient developed urosepsis. Ultrasound revealed acute onset right hydronephrosis with stone in renal pelvis. Urgent right nephrostomy was performed. After he recovered from this episode of acute infection, extracorporeal shock wave lithotripsy of right renal calculi was carried out. Computed tomography revealed cortical scarring of both kidneys. (Figure 3) Subsequently, ureteroscopy and laser lithotripsy of residual stones were carried out on both sides in two separate sessions. Results of urine and blood tests are given in Table 3.

**Table 3:** Results of laboratory investigations of Case 3

▪	Urea: 5.3 mmol/L.
▪	Creatinine: 121 umol/L.
▪	Haemoglobin: 117 g/L.
▪	July 2013: Urine protein: 1.43 g/L
▪	Protein:creatinine ratio: 201.4 mg/mmol.
▪	October 2013: Urine protein: 1.51 g/L.
▪	December 2013: Urine protein: 1.57 g/L.
▪	Urine protein from left nephrostomy: 0.52 g/24 hours;
▪	Urine protein from right nephrostomy: 0.53 g/24 hours.
▪	Serum total protein: 61 g/L; Albumin: 32 g/l.
▪	Serum IgG: 13.29 g/L (reference range: 6.00-16.00).
▪	Serum IgA: 2.85 g/L (reference range: 0.80-4.00).
▪	Serum IgM: 0.72 g/L (reference range: 0.50-2.00).
▪	Serum protein electrophoresis: No abnormal bands were detected.
▪	Serum Glomerular Basement Membrane Screen: Negative.
▪	Serum Neutrophil Cytoplasmic Antibodies Screen by fluorescence: Negative.

He was prescribed Ramipril 2.5 mg daily. At present, this patient does not have nephrostomy or ureteric stents. Blood pressure: 88/65 mm Hg. Urea: 6.5 mmol/L. Creatinine: 121 umol/L. Urine Protein: 0.33 g/L. Urine Protein: Creatinine ratio: 57 mg/mmol. He manages his bladder by penile sheath drainage and intermittent catheterisations. He lives in his home with his family and has been doing well.

## Discussion

### Proteinuria in spinal cord injury patients

The lesson from these cases is that health professionals should look for proteinuria in spinal cord injury patients with following risk factors: (1) those, who have not been taking anticholinergic drugs and at risk for developing vesicoureteric reflux and reflux nephropathy. (2) Patients, in whom vesicoureteric reflux has been demonstrated in video-urodynamics. (3) Patients with recurrent urine infection, hydronephrosis, renal scarring detected during imaging studies. (4) Patients with chronic infection – e.g. pressure sores, chronic osteomyelitis. (5) Longstanding spinal cord injury, although it is difficult to define a cutoff point, whether we should screen for proteinuria after ten years or twenty years. (6) Older patients. (7) Patients with co-morbidities like diabetes mellitus, hypertension.

In spinal cord injury patients, serum creatinine level may be low because of reduced muscle mass; estimated glomerular filtration rate may be misleadingly high. Serum creatinine level is not sensitive in detecting early deterioration of renal function in patients with spinal cord injury [6]. Kaji and associates [7] found serum creatinine to be within normal limits or only minimally elevated in spinal cord injury patients, despite significant reduction in creatinine clearance. Therefore, caution should be exercised while interpreting results of serum creatinine and eGFR in spinal cord injury patients.

### The optimal method for proteinuria detection in chronic spinal cord injury

Alshayeb and associates [8] carried out a retrospective analysis in 219 veterans with spinal cord injury, comparing Dipstick protein analysis and 24-h urine protein excretion. These researchers concluded that urine collections of 24-hour are still needed in the chronic spinal cord injury population for accurate detection of clinically significant proteinuria. Dipstick protein analysis may not reliably detect low-grade clinical proteinuria, whereas a urine protein: creatinine ratio below 0.3 may be used to rule out clinical range proteinuria.

### Significance of proteinuria in spinal cord injury patients

Spinal cord injury patients with proteinuria had more impaired renal function and increased mortality compared with spinal cord injury patients without proteinuria. Vaziri and associates [9] observed that 48% of 43 male hemodialysis patients with end-stage renal disease complicating spinal cord injury, exhibited nephrotic range proteinuria. Greenwell and associates [10] studied the predictive value of proteinuria and creatinine clearance in relation to mortality in patients with spinal cord injury. The presence of either proteinuria with protein of 500 mg/d or greater, or creatinine clearance less than 60 mL/min is associated independently with increased mortality in the chronic spinal cord injury population. The presence of both conditions further increases this risk.

### Proteinuria and progressive renal damage

Independent of the underlying causes, chronic proteinuric glomerulopathies have in common a sustained or permanent loss of selectivity of the glomerular barrier to protein filtration. Glomerular sclerosis is the progressive lesion beginning at the glomerular capillary wall, the site of abnormal filtration of plasma proteins. Injury is transmitted to the interstitium favoring the self-destruction of nephrons and eventually of the kidney. The results of experimental and clinical studies show that proteinuria may accelerate kidney disease progression to end-stage renal failure. Evidence indicates that this process occurs through multiple pathways, including induction of tubular chemokine expression and complement activation that lead to inflammatory cell infiltration in the interstitium and sustained fibrogenesis. Macrophages are prominent in the interstitial inflammatory infiltrate. This cell type mediates progression of renal injury to the extent that macrophage numbers in renal biopsy predict renal survival in patients with chronic renal [11].

### How to slow the progression of kidney disease in spinal cord injury patients with proteinuria?

Proteinuria is associated with a faster progression of kidney disease. In general, reduction in proteinuria correlates with slowing the progression of kidney disease. In controlled trials in Chronic Kidney Disease, Angiotensin Converting Enzyme (ACE) inhibitors and Angiotensin Receptor Blockers (ARB) reduce protein excretion by approximately 35% to 40%. In experimental animals, enhanced glomerular capillary pressure causes impaired glomerular permeability to proteins and permits excessive proteinuria. Reabsorption of filtered proteins can injure the tubular cells and interstitium of the kidney by activating intracellular events and the release of vasoactive and inflammatory mediators. Both ACE inhibitors and ARBs reduce the intraglomerular pressure and thereby, reduce the glomerular permeability barrier to proteins and limit proteinuria and filtered protein-dependent inflammatory signals [12].

### Guideline for prescribing angiotensin converting enzyme inhibitors or angiotensin receptor blockers in patients with proteinuria

National Institute for Health and Clinical Excellence [8] in England recommends ACE inhibitors/ARBs in patients with diabetes mellitus irrespective of whether they have hypertension or not, if albumin:creatinine ratio is > 2.5 mg/mmol (men) or > 3.5 mg/mmol (women).

If a patient does not have diabetes mellitus, but has hypertension and albumin: creatinine ratio < 30 mg/mmol, antihypertensive treatment should be instituted. In patients with hypertension and albumin: creatinine ratio ≥ 30 mg/mmol, Angiotensin Converting Enzyme inhibitors or Angiotensin Receptor Blockers should be prescribed [13].

In patients with albumin: creatinine ratio ≥ 70 mg/mmol with or without hypertension or cardiovascular disease, Angiotensin Converting Enzyme inhibitors or Angiotensin Receptor Blockers should be prescribed [13].

Therapeutic targets for ACE inhibitors and ARBs in chronic kidney disease are as follows [14]: (1) Blood pressure should be <130/80 mm Hg or lower in patients with spot urine total protein to creatinine ratio > 500–1,000 mg/g (56.8 – 113.0 mg/mmol). (2) Spot total protein to creatinine ratio should become less than 500–1,000 mg/g (56.8 – 113.0 mg/mmol).

### Lessons learnt from failure

Spinal injury patients with bladder involvement are well known to be at risk of high bladder residual volumes, urinary infections and stone formation. Over time these combined insults do take their toll on kidney function. While renal excretory function is often monitored by blood tests that measure urea and creatinine levels, proteinuria is not actively looked for. Proteinuria can occur when the blood tests are relatively normal, and should be acted upon as a sign of renal disease. Chronic kidney disease thus identified via blood or urine tests requires the same intensity of intervention as any other cause such as diabetes. In addition to the usual measures of correction of electrolyte and acid base imbalances, tight blood pressure control and cardiovascular risk reduction, spinal patients require added focus on prevention and early treatment of infections, and decompression of high pressure urinary systems. In those with more severe kidney damage, decisions should be made in advance with the patient on treatment ceilings and in those who are appropriate for dialysis, preparation for the same made in a planned manner so that their transition to renal replacement is smooth. Our paper highlights the need to detect proteinuria as marker for renal damage and the treatment of proteinuria as a means to defend renal function or at the very least, retard progression of renal disease.

We had been focusing on the urological care of these three patients; prescribing antibiotics for urine infection; providing drainage to obstructed kidneys; breaking urinary calculi by extracorporeal shockwave lithotripsy, percutaneous nephrolithotripsy, and ureteroscopic lithotripsy We did not routinely investigate these patients for proteinuria and co-existing renal disease until recently. We missed the opportunity for early detection of proteinuria and we failed to prescribe ACE inhibitors to slow the progression of kidney damage at an earlier stage.

### Take home message

(1) Spinal cord injury patients, who have not undergone video-urodynamics and not been taking antimuscarinic drugs are at risk for developing reflux nephropathy and proteinuria. (2) Patients who develop pressure sores, recurrent urosepsis, renal calculi, and hydronephrosis may develop proteinuria due to glomerulosclerosis. These patients should be tested for proteinuria during follow-up. (3) In the absence of other renal diseases causing proteinuria, spinal cord injury patients with significant proteinuria may be prescribed angiotensin-converting enzyme inhibitor or angiotensin-II receptor antagonist to slow progression of chronic renal disease and reduce the risk of cardiovascular mortality. (4) These spinal cord injury patients require careful monitoring of blood pressure and renal function while taking angiotensin-converting enzyme inhibitor or angiotensin-II receptor antagonist, as tetraplegic patients are likely to have low blood pressure, and some patients may have reduced glomerular filtration rate as well.

## Informed consent

The leading author wrote to three patients explaining the educational value of this case presentation and requested their permission to present their cases in the open access journal; the authors are most grateful to the patients for allowing us to include their case histories in this publication.

## Competing interests

The authors declare that they have no competing interests.

## Authors’ contributions

SV managed all cases, conceived the idea for this manuscript, collected the data, and wrote the draft. AA was the Consultant in Renal Medicine and provided expert advice. GS was the Consultant Urological Surgeon; BMS was Consultant in charge of three cases; PLH carried out radiological examinations. All authors have been involved in drafting the manuscript, revising it critically for important intellectual content, and have given final approval of the version to be published.
